# Restricted-Access Media Column Switching Online Solid-Phase Extraction UHPLC–MS/MS for the Determination of Seven Type B Trichothecenes in Whole-Grain Preprocessed Foods and Human Exposure Risk Assessment

**DOI:** 10.3390/toxics12050336

**Published:** 2024-05-06

**Authors:** Xiao Ning, Yongli Ye, Jian Ji, Yanchun Hui, Jingyun Li, Po Chen, Shaoming Jin, Tongtong Liu, Yinzhi Zhang, Jin Cao, Xiulan Sun

**Affiliations:** 1 School of Food Science and Technology, International Joint Laboratory on Food Safety, Synergetic Innovation Center of Food Safety and Quality Control, Jiangnan University, Wuxi 214122, China; nx200730079@163.com (X.N.); yyly0222@163.com (Y.Y.); jijian@jiangnan.edu.cn (J.J.); yinzhizhang@jiangnan.edu.cn (Y.Z.); 2 Key Laboratory of Food Quality and Safety for State Market Regulation, National Institute of Food and Drug Control, Beijing 100050, China; lijingyun@nifdc.org.cn (J.L.);; 3 Sanyo Fine Trading Co., Ltd., Beijing 100176, China

**Keywords:** trichothecene mycotoxin, UHPLC–MS/MS, prepared food, whole-wheat dumpling wrappers

## Abstract

With increasing health awareness and the accelerating pace of life, whole-grain prepared foods have gained popularity due to their health benefits and convenience. However, the potential risk of type B trichothecene toxins has also increased, and these mycotoxins in such foods are rarely regulated. In this study, a quantitative method combining a single-valve dual-column automatic online solid-phase extraction system with ultra-high-performance liquid chromatography–tandem mass spectrometry (UHPLC–MS/MS) was developed for the first time using restricted-access media columns. This method can simultaneously determine trace residues of seven type B trichothecenes within 15 min. The method is convenient, sensitive (limit of detection and quantification of 0.05–0.6 μg/kg and 0.15–2 μg/kg, respectively), accurate (recovery rates of 90.3%–106.6%, relative standard deviation < 4.3%), and robust (>1000 times). The established method was applied to 160 prepared food samples of eight categories sold in China. At least one toxin was detected in 70% of the samples. Whole-wheat dumpling wrappers had the highest contamination rate (95%) and the highest total content of type B trichothecenes in a single sample (2077.3 μg/kg). Exposure risk assessment indicated that the contamination of whole-grain prepared foods has been underestimated. The total health risk index of whole-wheat dumpling wrappers, which are susceptible to deoxynivalenol, reached 136.41%, posing a significant threat to human health. Effective measures urgently need to be taken to control this risk.

## 1. Introduction

Whole-grain prepared foods have gained widespread popularity due to their numerous health benefits, convenience, and diversity. However, whole-grain foods are usually less processed, they are more susceptible to fungal contamination, particularly by type B trichothecene toxins, represented by deoxynivalenol (DON). In 2020, European Food Safety Authority (EFSA) and in 2021, World Health Organization (WHO) both highlighted concerns about the presence of DON in cereals, especially noting whole-grain foods as significant sources of DON exposure. These reports emphasize the need for monitoring and managing the risk of fungal contamination in these food products to ensure consumer safety [1,2]. Although some countries have implemented regulations for DON, other known co–contaminating modified and emerging toxins have received little attention. In recent years, some studies have reported the individual toxicity of these derivatives, such as DON glycosylated (deoxynivalenol 3-glucuronide, D3G) or acetylated (3-acetyldeoxynivalenol, (3AcDON) and 15-acetyldeoxynivalenol (15AcDON)) derivatives, as well as their synergistic toxic effects with emerging toxins such as nivalenol (NIV). To date, comprehensive information on the composition and concentration of mycotoxins in prepared foods is very limited, highlighting the urgency of comprehensive monitoring and adequate risk assessment of mycotoxin contamination in these foods.

Liquid chromatography–tandem mass spectrometry (LC–MS/MS) analysis is the most powerful alternative analytical method for mycotoxins with different physicochemical properties, enabling sensitive, fast, and reliable multi–target analysis in food matrices [3,4]. However, the complex food matrix makes it crucial to perform sample pretreatment steps to pre–concentrate the target compounds and reduce matrix effects. Solid–phase extraction (SPE) is a popular method for preparing complex samples [5]. Compared with traditional offline SPE, online SPE coupled with UHPLC–MS/MS contributes to the automation of the analytical process. To our knowledge, the currently reported SPE–LC–MS methods for simultaneous analysis of multiple trichothecenes are all offline modes, which are cumbersome, time–consuming, and labor–intensive, especially when dealing with a large number of samples [6]. Therefore, establishing an automated online SPE–UHPLC–MS/MS method will significantly improve the efficiency of analysis, reduce solvent consumption, and minimize environmental pollution. Unfortunately, due to the ease of SPE column clogging caused by macromolecules such as proteins in the samples, this robustness defect severely hinders the large–scale application of online automated mode to food samples [7]. Restricted access media (RAM) provides an effective approach to address this limitation. The chromatographic column packing simultaneously bonds hydrophilic groups with steric hindrance and hydrophobic groups for retention. Through two separation principles, it effectively prevents large molecules (such as proteins) from entering the adsorbent and causing analyte retention. To date, RAM chromatographic columns have not been applied to trace analysis of mycotoxins in food.

This study proposed an automated online SPE–UHPLC–MS/MS method using RAM columns for the simultaneous quantitative analysis of seven type B trichothecenes in whole-grain prepared foods. The method was validated in three sample matrices, including instant brown rice, oatmeal, and whole–wheat flour products, and the results met the acceptable criteria specified in European Commission Decision (EC) No. 2002/657/EC [8]. The proposed method is not only sensitive, accurate, and easy to operate but also successfully applied to analyze 160 actual samples collected from the Chinese market, covering eight different categories, including brown rice, whole-grain infant rice flour, oatmeal, instant oats, oatmeal cookies, whole–wheat bread, whole–wheat noodles, and whole–wheat dumpling wrappers. Finally, the exposure risk of these prepared foods susceptible to mycotoxin contamination was assessed by calculating the hazard quotient (HQ) and hazard index (HI). This is the first study on the analysis of trichothecenes and human exposure risk assessment in whole-grain–based prepared foods, providing a powerful tool and valuable reference for protecting consumers from the potential health risks of mycotoxin contamination.

## 2. Materials and Methods

### 2.1. Chemicals and Reagents

Acetonitrile (ACN), mass spectrometry grade, was purchased from Anple Laboratory Technologies Co., Ltd. (Shanghai, China); acetic acid (HAc), chromatographic grade, was provided by J&K Scientific Ltd., (Beijing, China); water was ultrapure water prepared by MilliQ. Seven standard solutions, each at a concentration of 100 μg/mL, were obtained from Romer Labs (Tulln, Austria), including DON, 3AcDON, 15AcDON, D3G, NIV, fusarenon-X (FusX), and de-epoxy-deoxynivalenol (DOM-1). Additionally, 180 whole-grain prepared food samples were purchased from the Chinese market. All chromatographic measurements were performed on a NASCA II UHPLC system (OSAKA SODA, Osaka, Japan), which consists of a NASCA II all-in-one machine (including a degasser, dual pumps, an autosampler, and a heating kit). Additionally, an external F3012 six–port valve, an F3202 degasser, and an F3301 dual pump form a two-dimensional liquid chromatography system connected to a QTRAP 5500 triple quadrupole-linear ion trap mass spectrometer (AB SCIEX, Toronto, SD, Canada). AB SCIEX Analyst chromatography software (1.5 version) was used for data acquisition and analysis. The pretreatment solid–phase extraction was carried out using a CAPCELL PAK MF C8 SG80 chromatographic column (5 μm, 150 mm × 2 mm, OSAKA SODA, Japan) with a pressure limit of 20 MPa. A CAPCELL PAK C18 MGII (2 μm, 100 mm × 2 mm) analytical column (OSAKA SODA, Japan) was used for chromatographic separation; a balance (METTLER TOLEDO, Greifensee, Switzerland); Fungilab ultrasonic device (FUNGILAB, Madrid, Spain); and Milli–Q UltrapUre Ion–Ex–TM ultrapure water system (MILLIPORE, St. Louis, MO, USA) were also used.

### 2.2. Preparation of Standard Solutions

The selection of concentrations for the mycotoxin standard solutions was based on their sensitivity to the instrument and their co-stability in the standard solutions. The mixed standard solution contained 2 μg/mL of 3AcDON, FusX, and NIV; 4 μg/mL of DON and DOM-1; 0.4 μg/mL of D3G; and 8 μg/mL of 15AcDON. All solutions were prepared in acetonitrile, stored in the dark at –20 °C, and diluted with the initial solvent for SPE–UHPLC–MS/MS analysis.

### 2.3. Online SPE–LC–MS/MS Method

#### 2.3.1. Mass Spectrometry Conditions

The ion source was a Turbo–V electrospray ion source, operating in negative ion mode with a spray voltage of –2400 V, ion source temperature of 350 °C, spray gas (GAS1) at 25 psi, drying gas (GAS2) at 25 psi, and curtain gas at 40 psi. Multiple reaction monitoring (MRM) mode was used to monitor the analytes, with an entrance potential (EP) of –10 V, collision cell exits potential (CXP) of –15 V, and declustering potential (DP) of –100 V. The *m/z* values of the precursor and product ions for each substance, as well as the collision energy (CE) parameters in multiple reaction monitoring (MRM) mode, are shown in Table 1.

#### 2.3.2. Liquid Chromatography Analysis

**Figure 1:**
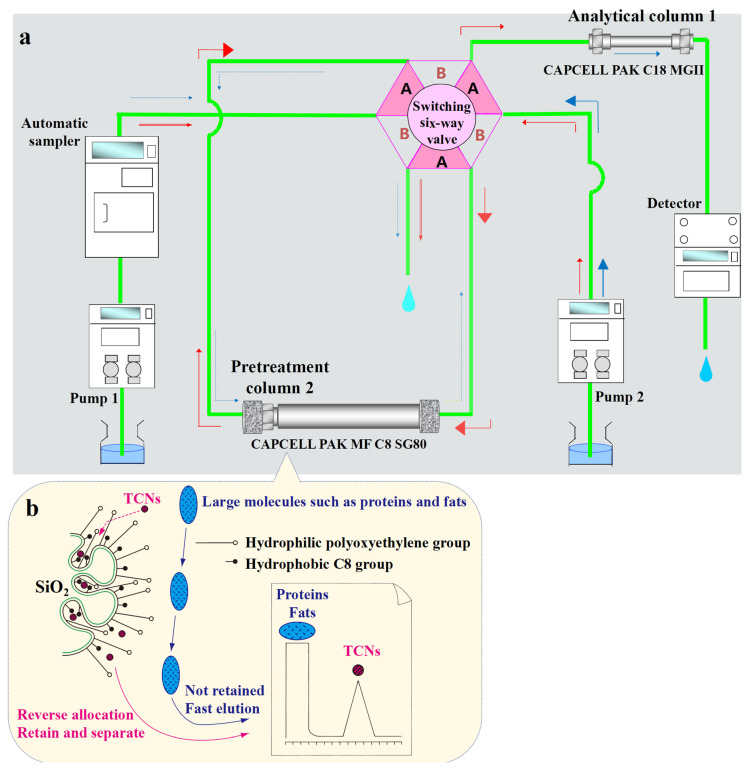
Schematic of the automated online SPE–HPLC system (**a**), and the extraction and clean–up principle of a RAM column (**b**).

The principle of the online SPE–LC–MS/MS method is shown in Figure 1a, and the gradient distribution of the two pumps and the corresponding positions of the six-port valve is shown in Table 2. At a low proportion of the organic phase, from 0 to 1.3 min, large molecular compounds such as proteins, fats, and starches that may be present in the sample are eluted, and the target substances are retained. At this time, the two-dimensional system balances the analytical column (column 2) through pump 2. From 1.3 to 11.1 min, after switching the six-port valve, pump 2 performs reverse flushing of column 1 and enters column 2, and the target substances enriched in column 1 enter pump 2 with the gradient conditions of the two–dimensional mobile phase to start the two-dimensional analysis process.

Chromatographic columns: One-dimensional: CAPCELL PAK MF C8 SG80 (2 mm i.d. × 150 mm, particle size 5 μm) (OSAKA SODA, Japan); two-dimensional: CAPCELL PAK C18 MGII (2 mm i.d. × 100 mm, particle size 2 μm) (OSAKA SODA, Japan). The column temperature was 25 °C, and the injection volume was 10 μL.

### 2.4. Samples and Sample Preparation

The method development and validation were conducted using three types of sample matrices: blank instant brown rice (*n* = 6), oatmeal (*n* = 6), and whole-wheat flour products (*n* = 6) collected from the Chinese retail market. All collected samples were ground into fine powder using a laboratory mill to achieve sufficient homogeneity and stored in sealed plastic bags in a dark environment at 4 °C until analysis.

After the method was validated to meet the acceptable criteria specified in European Commission Decision (EC) No. 2002/657/EC [8], it was applied to analyze 180 actual samples collected from the Chinese retail market, including brown rice, whole-grain infant rice flour, oatmeal, instant oats, oatmeal cookies, whole–wheat bread, whole–wheat noodles, and whole–wheat dumpling wrappers.

#### Extraction Procedure

Weigh 2.0 g of whole-grain prepared food. Add 10 mL of FA–ACN–water (1:84:14, *v/v*) mixture, vortex for 30 s, and extract by ultrasound for 10 min. After centrifugation (9000 r/min) for 5 min, pass the supernatant through a 0.22 μm filter membrane and set aside.

### 2.5. Method Validation

The optimized method was validated according to the existing procedures in [8,9]. The method can be used to analyze seven TCNs in nine types of samples, including cereal bars, oatmeal, whole-wheat bread, whole-wheat biscuits, whole-wheat steamed buns, whole-wheat noodles, instant rice, whole-wheat dumpling wrappers, and infant cereal rice flour. The performance characteristics evaluated include linearity, limit of detection (LOD), limit of quantification (LOQ), selectivity, matrix effect, recovery, repeatability, reproducibility, and robustness (details were described in the Supplementary File).

### 2.6. Risk Assessment

Given the severe toxicity of the target mycotoxins, it is crucial to assess their potential exposure risk to human health in whole-grain-based prepared foods by evaluating the contamination levels of the seven target mycotoxins and combining them with relevant food consumption data. The estimated daily intakes (EDIs) and hazard quotient (HQ) values of the mycotoxins were calculated using the following formulas:

EDI (μg/kg bw/day) = (C × F)/bw

where, C is the average contamination concentration (μg/kg) of type B trichothecenes in whole-grain prepared food samples, and F is the average consumption (g/day) of whole-grain foods. As there are currently no authoritative statistics on the consumption data of whole-grain prepared foods in China, this study adopted the recommended daily intake value of whole grains for adults in the Chinese Dietary Guidelines, which is 100 g (the average of 50–150 g), as the food factor (F value) for estimating exposure [10]. bw is the human body weight (kg), and the default international average body weight for adults is 60 kg [11].

The EFSA has set tolerable daily intakes (TDIs) to assess the exposure risk of mycotoxins in food under controllable conditions. The TDI for DON is 1 μg/kg·day·bw, and the TDI for NIV is 0.7 μg/kg·day·bw [12,13]. Due to the limitations of toxicity studies on D3G, 3AcDON, 15AcDON, and FusX, there are currently no officially confirmed TDIs for these compounds. In 2017, EFSA conducted a comprehensive risk assessment of DON and its derivatives (including D3G, 3AcDON, and 15AcDON). The report supported the view of the Joint FAO/WHO Expert Committee on Food Additives, which states that the intake of 3AcDON and 15AcDON should be fully counted as DON equivalents, while 30% of the D3G intake should be counted as DON equivalents, and the TDI value of DON should be applied for risk assessment [14]. FusX may have similar toxic effects to NIV, and the TDI value of NIV can be used as a reference for risk assessment [15]. The hazard quotient (HQ) is usually introduced to represent the risk level of dietary intake of each toxin, which is calculated as the ratio of EDI to TDI, as shown in the following formula:

HQ (%) = (EDI/TDI) × 100

An HQ < 100% is considered an acceptable dietary exposure level for mycotoxins and does not pose a health threat to humans, while an HQ > 100% indicates that the dietary exposure level exceeds the permissible limit and poses a health threat and will therefore be considered a serious safety issue [16].

Furthermore, since the analyzed TCNs involve DON and NIV or their derivatives, and the interaction mechanisms between each toxin are not yet clear, the total risk can be estimated by directly combining the HQ values of each toxin and calculating the hazard index (HI, %) using the following formula:

HI (%) = ∑ HQ i, where i ∈ {DON, D3G, 3AcDON, 15AcDON, NIV, FUsX, DOM-1}

An HI > 100% for multiple mycotoxins indicates that dietary exposure may have a significant adverse effect on human health.

### 2.7. Data Analysis

When the mycotoxin levels detected in the samples were higher than the LOQ, the samples were considered positive, while samples with contamination levels lower than the LOQ were considered negative.

During the dietary risk assessment, mycotoxins that were not detected or below the LOQ were considered to be half of the LOD (LOD/2).

Statistical analysis was performed using SPSS software (version 22.0, IBM Corp., Armonk, New York, NY, USA) to calculate correlation coefficients (R2 ≥ 0.99). OriginPro software (2019b, OriginLab Inc., Northampton, MA, USA) was used to plot the spectra.

## 3. Results and Discussion

### 3.1. Selection of Sample Extraction

**Figure 2:**
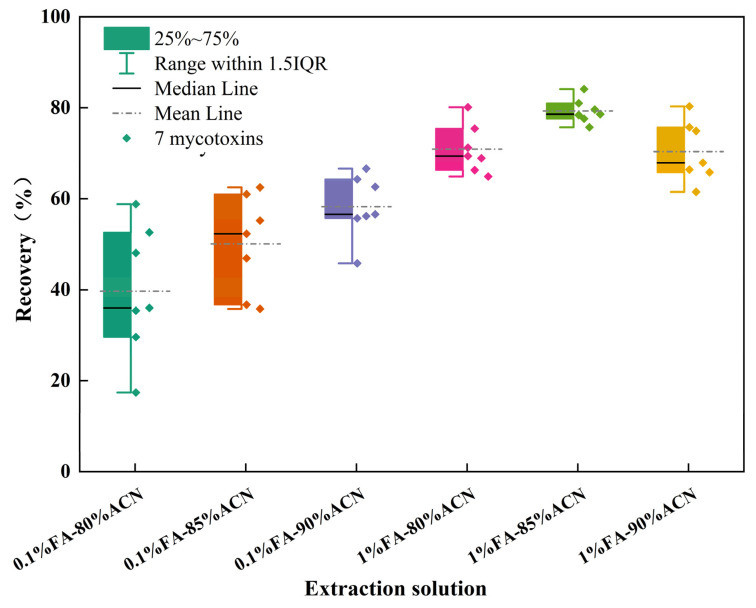
Average recoveries of 7 B-type trichothecenes extracted by 6 solutions.

It is necessary to select a suitable extraction solvent for mycotoxins. Studies have shown that type B trichothecenes are highly soluble in organic solutions due to their lipophilic and hydrophobic properties [17]. The addition of a small amount of water promotes the wetting of the sample matrix and facilitates the penetration of organic solutions into the food. Furthermore, organic acids can disrupt the tight binding between the analytes and other food nutritional components (such as proteins and sugars), thereby promoting the extraction of mycotoxins [18]. Compared to methanol/water mixtures, the use of acetonitrile/water mixtures can effectively reduce the co-extraction of interfering substances from the samples and achieve satisfactory recoveries [19,20]. Therefore, this study compared the extraction effects of acetonitrile aqueous solutions containing 0.1%–1% formic acid. As shown in Figure 2, the mean recoveries of the seven mycotoxins extracted with six solutions were 39.7%, 48.2%, 58.3%, 70.9%, 79.3%, and 70.4%, respectively. Although none of the six solvents achieved ideal recoveries for all analytes after extraction without purification, it is worth noting that the analyte recoveries extracted with 1% formic acid–85% acetonitrile aqueous solution were tightly distributed in the box plot, with a median quartile (Q2) of 78.6% and a third quartile (Q3) of 80.3%, showing the relatively best extraction effect. Therefore, this solvent was chosen as the optimal extraction solvent for further purification and analysis.

### 3.2. Optimization of UPLC–MS/MS

Developing a suitable sample purification method is often considered the critical first step in the entire analytical chain, as it is the most time-consuming and error-prone step [21]. The RAM chromatographic column plays a key role in the online SPE analysis of complex food samples through two separation principles. As shown in Figure 1b, a uniform thin film coated with siloxane polymers is formed on the surface of high-purity silica gel. Simultaneously, hydrophilic groups with steric hindrance (polyoxyethylene) and hydrophobic groups (C8) for retention are bonded. Large matrix molecules such as proteins are affected by the steric hindrance of hydrophilic groups and the size exclusion effect caused by small pore size, and they are not retained but eluted directly at the dead time. In contrast, the target small molecule mycotoxins can interact with the hydrophobic groups to achieve retention and separation. In this study, we also attempted to use a CAPCELL PAK MF Ph–1 SG80 chromatographic column (4.6 mm × 150 mm, id, 5 μm, OSAKA SODA, Japan) with phenyl as the hydrophobic group for pretreatment. The retention capacity of the MF Ph–1 column was weaker than that of the C8 column, with NIV and D3G having retention times of 1.63 min, close to the dead time (1.2 min) at that point, which may cause loss during pretreatment valve switching and affect quantitative results. Therefore, it was abandoned.

To optimize the mass spectrometry method, a standard solution of a single compound was injected into the LC–MS/MS system. Analyses were performed in positive/negative ion mode, and the two highest-intensity ions observed for each analyte were used as Quantifier and Qualifier, respectively. When the mobile phase used 0.1% formic acid (FA) or 0.1% HAc as the aqueous phase, the corresponding precursor ions were [M + H]^+^ or [M+CH_3_COO]^−^, respectively. Compared to positive ion mode, there was a trend of increased response intensity of analyte ions in negative ion mode, with increases ranging from 1.7-fold (D3G) to 6.0-fold (3AcDON). It can be seen that the [M+CH_3_COO]^−^ provided by HAc greatly enhanced the ionization of the analytes. When the HAc concentration was reduced from 0.1% to 0.01%, the signal intensity of 3AcDON and others increased by more than 1.8 times. Further reducing from 0.01% to 0.005%, all signal intensities continued to increase except for DON and FusX. However, the use of 0.005% HAc resulted in poor precision for all analytes, with the RSD range of peak areas for the tested mycotoxins (*n* = 3) between 11% and 32%. When the HAc concentration was 0.01%, the RSD was between 1% and 4%. Therefore, 0.01% HAc aqueous solution was selected as mobile phase (A), with ACN as mobile phase (B), and all compounds had good chromatographic peak shapes.

**Figure 3:**
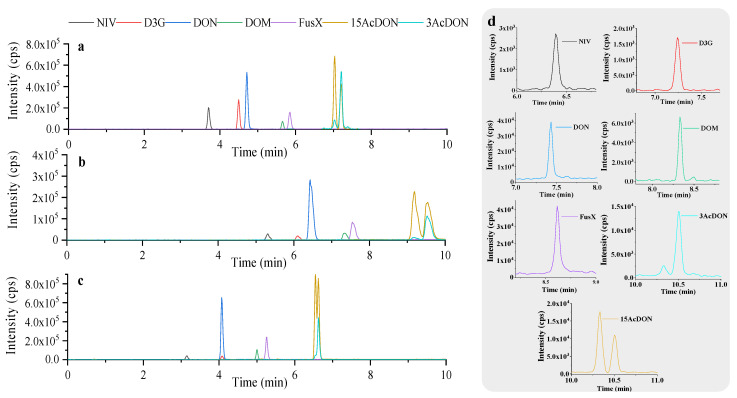
The total ion chromatogram was obtained by the CAPCELL PAK C18 MGII column (**a**), the CAPCELL CORE C18 column (**b**), or the ADME–HR column (**c**). Extracted ion chromatograms of 7 analytes obtained by linking CAPCELLPAK MF C8 SG80 with CAPCELLPAK C18 MGII under optimal conditions (**d**).

On this basis, the chromatographic separation conditions were further optimized to achieve satisfactory separation within a short analysis time. DON derivatives have similar structures and properties, especially the positional isomers 3AcDON and 15AcDON, which exhibit common precursor ions (*m/z* 397.3) and similar product ions. Therefore, baseline separation through gradient elution is necessary to achieve accurate quantification. In this study, key parameters were explored in terms of column type, flow rate, and gradient. As shown in Figure 3a, CAPCELL PAK C18 MGII is a moderately polar C18 column. At a flow rate of 0.25 mL/min, a mild gradient of 25–30% B was maintained for 5–8 min for elution, achieving baseline separation of 3AcDON and 15AcDON. Further research found that the CAPCELL CORE C18 column is a core-shell column with C18 as the bonded phase. The packing of core-shell columns has a solid core–porous surface structure, which, compared to fully porous packing, can achieve good retention and separation within a shorter analysis time, improving analytical efficiency. However, when using the CORE C18 column as the analytical column, the results are shown in Figure 3c. The analysis time was shorter than that of the MGII column, and the separation of 3AcDON and 15AcDON could not meet the analytical requirements, so it was abandoned. The ADME–HR column has a bonded phase of a sterically caged adamantyl group, a non-linear bonded phase. Due to the structural difference from the linear bonded phase, this column has ultra-high surface polarity while possessing certain surface hydrophobicity, making its retention effect on polar compounds and compounds containing polar groups superior to C18 columns, which is conducive to the separation of isomers. Therefore, we also tried using the ADME–HR column as the analytical column, but the results are shown in Figure 3b. Although the separation of 3AcDON and 15AcDON was better on this column, the peak shape was poor (peak too wide), so it was abandoned. Finally, the CAPCELL PAK C18 MGII was selected as the analytical column since after pretreatment with a front–end RAM column, quantitative analysis of seven compounds can be simultaneously performed within 11 min of a single injection. The extracted ion chromatogram is shown in Figure 3d.

In a study, the toxicokinetics of DON, and its acetylated derivatives 3AcDON and 15AcDON, and its main metabolite DOM-1 in chicken and pig plasma were determined by LC-MS/MS after a simple deproteinization step with acetonitrile, followed by evaporation and reconstitution [22]. The method focuses on the analysis of these mycotoxins in animal plasma. However, the difference in sample matrices presents distinct challenges for sample preparation. In contrast, our method utilizes an online SPE using a RAM column for the cleanup of complex cereal-based food matrices. The RAM column allows for the direct injection of samples by trapping small analytes while eluting macromolecules, effectively removing potential interferences from the matrix.

Furthermore, our method achieves satisfactory separation of the analytes, especially the positional isomers 3AcDON and 15AcDON, within a short analysis time using a core-shell analytical column (CAPCELL PAK C18 MGII). Although the two methods are designed for different sample matrices, both demonstrate good performance for the quantitative analysis of DON and its derivatives. Our approach, tailored for cereal-based prepared foods, offers advantages in terms of efficient sample cleanup and enhanced chromatographic separation, making it suitable for the reliable determination of type B trichothecenes in these complex matrices.

### 3.3. Time of Valve Switching

During 0–1.3 min, the six-port valve is in position A, and the RAM column is performing sample pretreatment. According to the flow rate, column inner diameter, and column length, the system’s dead time is calculated to be approximately 1.0 min. Since we want to remove large molecules from the sample through online pretreatment, and these substances are not retained on the RAM column, while also ensuring that the target small molecules can be retained on the RAM column without being eluted, we choose to complete the pretreatment in as short a time as possible and quickly switch the retained target substances to the two-dimensional system to ensure that their recovery is not compromised. Based on the above considerations, we switch the six-port valves to position B at 1.3 min. During 1.3 min–11.1 min, the RAM column is connected in series with the analytical column in the two-dimensional system, and the RAM column adopts a reverse-phase flushing mode (opposite to 0–1.3 min) to backflush the target substances retained on the RAM column after pretreatment to the analytical column (which is in forward-flush mode) for sample separation and detection. After all target components have been effectively analyzed, the six-port valve is switched back to position A at 11.1 min and maintained until 15 min to balance the system and prepare for the analysis of the next sample. The valve switching times are shown in Table S1.

### 3.4. Method Validation

In this study, the gradient elution program was carefully designed to prevent this possibility, and no carry-over effects were observed for any compounds. The RAM column can be used to extract multiple samples without significant signal loss or the need for replacement. All steps of method optimization, validation, and sample analysis were performed using the same RAM column, with a total of over 1000 injections.

The established SPE–UPLC–MS/MS method is suitable for the quantification of seven type B trichothecenes in whole-grain prepared foods. Therefore, appropriate sensitivity assessment is necessary. As shown in Table 2, with instant brown rice, oatmeal, and whole-wheat flour products (whole-wheat bread) as representative matrices, the LODs and LOQs of the target toxins were 0.05–0.6 µg/kg and 0.15–2 µg/kg, respectively. In the last decade, the target mycotoxins in cereals reported using LC–MS/MS are generally in the range of 0.02–50 μg/kg for LODs and 0.11–200 μg/kg for LOQs [9,22,23,24,25,26,27], as detailed in Table S2. In comparison, this study established an online automated system that completes purification and enrichment in one step, and the method is sensitive enough to meet the analytical requirements for trace residues of seven trichothecenes, such as DON, in prepared cereal foods.

According to the SANTE guidelines [28], matrix effects (MEs) in the range of −20% to +20% are considered insignificant. As shown in Table 2, matrix effects varied among different compounds, with an overall fluctuation range of –14.4% to 5.4%, but early–eluting compounds appeared to exhibit higher matrix effects than later–eluting compounds. Consistent with previous literature assessments, DON, D3G, and NIV commonly exhibit varying degrees of matrix effects in LC–MS/MS analysis, especially in oat matrices, mainly manifesting as ion suppression. Among them, D3G and NIV are more significantly affected by matrix effects than DON [29]. To reduce the impact of matrix effects, methods such as isotope-labeled internal standards, matrix-matched standard curves, dilution and injection can be adopted. Considering that the pretreatment method provided in this study has greatly reduced the matrix effects, which are not as significant as reported in the above literature (exceeding the range of −20% to +20%), a matrix-matched calibration strategy that balances cost and sensitivity was adopted to minimize matrix effects and ensure accurate quantification. The linear regression data are summarized in Table 2. All relevant mycotoxins had r values greater than 0.999 within the applicable working range, indicating good linearity of the calibration curves.

The intra-day and inter-day precision (expressed as % RSD) in three typical sample matrices met international standards [30,31], as shown in Table S2, with values below 4.29 and 11.7%, respectively, which are acceptable for complex samples [32,33]. At low concentrations (the first point of the calibration curve), the RSD values for the seven compounds were less than 9.5%, indicating good precision of the method, mainly due to the automated control of the online analytical system and the robustness of the RAM column. The accuracy of the method was evaluated through recovery experiments using spiked samples at three concentrations. The recoveries ranged from 89.7% to 103.6% (Table S3). It is noteworthy that the proposed method can achieve good precision and accuracy without the use of isotope-labeled internal standards, which is important in reducing the generation of hazardous waste and greatly reducing funding investment costs.

Quality control (QC) samples were prepared in the initial mobile phase (standard concentration was 10 times the quantification limit of each analyte) and analyzed before and after each batch of samples. After the last QC sample, acetonitrile was injected to clean the system and check for any residual effects before starting the analysis of a new batch of samples. No carry-over effects were observed.

### 3.5. Application to Real Samples

#### 3.5.1. Occurrence of Mycotoxins in Samples

**Figure 4:**
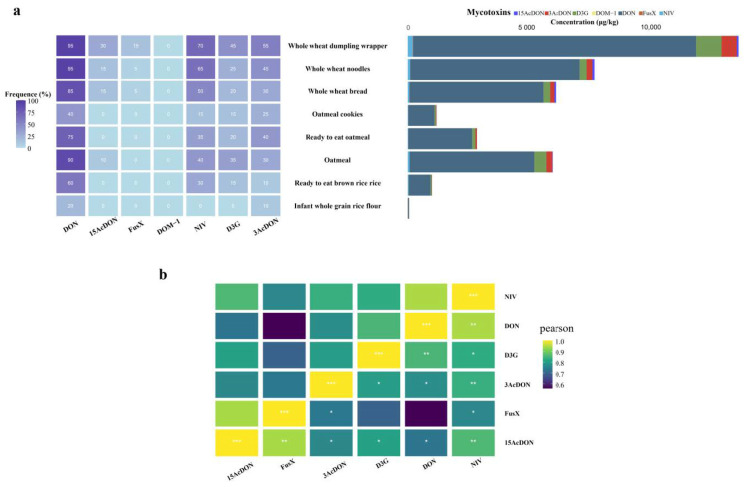
Occurrence of B-type TCNs in 160 whole-grain prepared foods collected across China (**a**). Co–contamination heatmap of the Pearson correlation matrix indicating the prevalence of B-type TCNs combinations (**b**). The color gradient ranges from dark purple to light yellow, corresponding to correlation values from 0.6 to 1.0, respectively. Asterisks indicate statistical significance (* *p* < 0.05, ** *p* < 0.01, and *** *p* < 0.001).

Application, the established method was used to detect seven type B trichothecene toxins in 160 whole-grain prepared food samples of eight categories collected from the Chinese market. The levels of the seven mycotoxins were obtained, and the results are shown in Figure 4 and Table S3. Whole-grain products retain the germ and bran of the grain and have not undergone deep processing such as peeling and grinding, resulting in a low toxin removal rate. Among the 160 samples, 112 (70.0%) contained at least one toxin. DON had the highest detection rate (70.0%) and the highest maximum detected concentration (1685.2 μg/kg). This residue level is compliant with the Codex Alimentarius Commission standard (2000 μg/kg) and is slightly lower than the European Union (EU) standard (1750 μg/kg). However, it exceeds the standard set by China (1000 μg/kg) [34,35,36]. NIV, 3AcDON, D3G, and 15AcDON followed, with detection rates of 38.1%, 30.6%, 21.9%, and 8.8%, and maximum detected concentrations of 90.3, 208.1, 28.6, and 47.2 μg/kg, respectively. This result is supported by multiple studies, as DON is usually the trichothecene toxin with the highest detection rate and contamination level in cereal grains such as wheat and oats and their processed products [37,38]. Compared to other trichothecenes such as NIV, *F. graminearum* has a higher capacity to biosynthesize DON, with a faster production rate per unit of fungal biomass. This higher biosynthetic efficiency, coupled with the rapid growth of *F. graminearum* on cereal grains, results in higher levels of DON contamination [39]. Acetylated DON derivatives (3AcDON and 15AcDON) originate from the secondary metabolic processes of specific chemotypes of *F. graminearum* and usually co-contaminate grains with DON, but at relatively lower levels [40]. It is worth noting that the detection rate and content of 3AcDON are usually higher than those of 15AcDON [41], which is confirmed again by the results in this study. In comparison, although the contamination levels of D3G and NIV are lower than those of DON, they should not be ignored. Several studies have demonstrated that grains can be co-contaminated with NIV and D3G, with concentrations ranging from tens to hundreds of μg/kg [42,43]. This suggests that when assessing the risk of trichothecene contamination in cereal products, in addition to focusing on free DON, attention should also be paid to other coexisting analogs [44].

FusX, as an acetylated derivative of NIV, exhibits relatively low toxicity, and current research primarily focuses on the detection of this toxin in grains, which are raw materials for processed foods. A study conducted in Canada from 2008 to 2010 demonstrated that FusX was detected in less than 1% of wheat samples, with the highest concentration being only 30 μg/kg [45]. In contrast, analyses by Bryła and his team of Polish grain samples from 2014 to 2016 found detection rates of FusX in wheat and barley to be 4% and 2%, respectively, with the highest concentration reaching 122 μg/kg [46]. Although these values are higher than those reported in Canada, they remain low compared to the primary trichothecene DON. Similarly, in this study, FusX was detected in 5 out of 160 samples, with the highest concentration found in whole-wheat dumpling wrappers being 17.9 μg/kg. However, a study in Sichuan Province, China, revealed a significantly higher detection rate of FusX in rice samples, at 28.6%, with the highest concentration detected being 455.1 μg/kg [47]. These findings indicate that while the contamination rate of FusX in grains is generally low (less than 5%), the detection rate and level of contamination vary according to region and crop type. Given that contamination in raw materials can migrate to the final processed products, continuous monitoring of FusX contamination levels in grains is crucial.

DOM–1 is a de-epoxy derivative of DON. This method monitored its contamination in 160 whole-grain-based foods, and the results showed no detection. DOM–1 mainly originates from the metabolic transformation of DON in animals and is rarely directly detected in grains and their processed products. Broekaert et al. [48] found that the proportion of DOM–1 detected in 190 samples (grains such as wheat, corn, and oats) was only 2.1%, with a maximum detected concentration of 23 μg/kg, which is much lower than DON and other derivatives. Moreover, Payros et al. [49] analyzed various DON derivatives in wheat and its flour products, and DOM–1 residue was not detected in any of the samples. This study further confirms that the risk of direct contamination of this derivative in grains is low.

#### 3.5.2. Contamination Characteristics of Type B Trichothecenes in Different Types of Samples

From Figure 4a, it can be seen that TCNs contamination varies greatly among different food categories. Whole-wheat dumpling wrappers are the most severely contaminated prepared food, followed by whole-wheat noodles, oatmeal, and whole-wheat bread. In contrast, infant whole-grain rice flour is almost uncontaminated, while instant brown rice, instant oats, and oatmeal cookies have lower contamination levels. The reasons for this difference may include the following: First, the quality and contamination status of raw grains differ. Cereals such as wheat and oats are more susceptible to *F. graminearum* infection, while rice has a relatively stronger resistance to infection [50]. Second, product quality standards and regulatory efforts vary. As a special dietary food, infant food has stricter raw material quality control and a higher frequency of self-inspection by enterprises and government supervision, and it is difficult for highly contaminated products to enter the market. Third, there are differences in food processing techniques. High-temperature baking, extrusion puffing, and cooking processes help reduce toxin levels in foods such as oatmeal cookies and instant oats [51]. The moisture content of dumpling wrappers is higher than other cereal products such as bread and biscuits (usually around 35%), and DON has good solubility in moisture, making it easier to accumulate in high-moisture dumpling wrappers. The processing of dumpling wrappers is relatively complex, from dough mixing and resting to rolling and shaping, which takes a long time. The lengthy processing provides more opportunities for residual Fusarium to multiply and produce toxins [52]. Finally, most dumpling wrappers sold in the market are in bulk, and improper storage conditions during the shelf life can easily lead to moisture absorption and mold growth. Residual Fusarium will multiply in large quantities, further increasing the DON content. In addition, the pH value of dumpling wrappers (usually between 5.0–6.0) provides favorable acidic conditions for the stability of DON [53].

Therefore, to reduce DON contamination in whole-wheat dumpling wrappers, it is necessary to control the entire chain from farm to table, improve the quality of whole-wheat flour raw materials, optimize production processes, improve storage and transportation conditions of finished products, and take multiple measures simultaneously to achieve good results.

#### 3.5.3. Co-Contamination Patterns

In the whole-grain prepared food samples in this study, the main contamination patterns of DON + NIV, DON + D3G, and DON + NIV + 3AcDON (Figure 4) are all produced by Fusarium fungi such as *F. graminearum* and *F. culmorum*. It has been confirmed that the biosynthesis of DON and NIV is regulated by the same gene cluster (Trichothecene biosynthesis gene cluster, TRI) [54]. Mutations or deletions of the TRI8 and TRI13 genes in the gene cluster lead to *F. graminearum* synthesizing different types of DON derivatives (3AcDON and D3G) [55], which is further demonstrated by the results of this study. In addition, climate change and global warming have led to more extreme weather conditions with high temperatures and heavy rainfall, providing favorable conditions for the growth and reproduction of Fusarium fungi, which also exacerbates the risk of toxin contamination in these food raw materials.

### 3.6. Risk Assessment

Considering that DOM–1 was not detected in any of the samples, and infant whole-grain rice flour was almost uncontaminated, we proceeded to conduct a risk assessment for the residues of six trichothecene mycotoxins (TCNs), excluding DOM-1, in seven other categories of whole-grain prepared foods. The hazard quotient (HQ) and hazard index (HI) were calculated using the method recommended by the WHO, acknowledged for its global applicability and comparative risk assessment across various foodstuffs.

As shown in Table 3, whole-wheat dumpling wrappers had the highest average contents of DON and D3G, reaching 590.77 μg/kg and 52.70 μg/kg, respectively. The exposure level (EDI) of DON in whole-wheat dumpling wrappers was 0.9846 μg/kg·day·bw, close to its toxicity reference value (TDI) of 1 μg/kg·day·bw, with a health risk quotient (HQ) as high as 98.462%. Whole-wheat dumpling wrappers also had the highest total health risk index (HI), reaching 136.408%, indicating that the total risk of type B trichothecenes in this food has exceeded the standard. The risk of DON has been previously found in commercially available noodles, steamed buns, and flour in China [56,57,58], and this study is the first to find it in whole-grain prepared foods, reflecting that DON contamination in whole-grain prepared foods remains a serious food safety issue that urgently needs effective measures to control. The DON contents in oats, instant oats, and oatmeal cookies were relatively low, but still reached 260.36, 133.93, and 55.86 μg/kg, respectively. The HQ value of oats was 43.393%, and the HI value was 60.419%, which also requires attention. In contrast, instant brown rice had the lowest contents and risk levels of various type B trichothecene toxins, and the overall risk was controllable. In addition to DON and D3G, 3AcDON and 15AcDON were co-contaminated in whole-wheat dumpling wrappers and whole–wheat noodles. Although the HQ values were not high, considering that existing studies have found synergistic toxic effects of these acetylated derivatives with DON in in vitro and in vivo experiments such as intestinal epithelial cells, piglets, and zebrafish [59,60,61], their hazards should not be ignored. However, it is pertinent to acknowledge that this additive approach may not always precisely reflect the interactive complexity of mycotoxin exposure.

## 4. Conclusions

For the first time, this study established an online SPE–UPLC–MS/MS method for the rapid quantitative determination of seven type B trichothecene toxins in whole-grain prepared foods. By designing a single-valve dual-column system and using a high-pressure six-port valve to connect the RAM purification column and the analytical column in parallel, column switching after sample injection was achieved, simultaneously accomplishing purification and separation. The results showed that this method improves efficiency and speed on the basis of reducing costs (less manual input and no need for internal standards) and has good robustness. After validation, the method was found to be sensitive and accurate, suitable for routine large-scale quantitative analysis of type B trichothecene toxin contamination in whole-grain prepared foods. The practicality of the method was verified by quantifying seven mycotoxins in 160 batches of eight categories of whole-grain prepared foods.

Overall, the total risk of type-B trichothecenes in whole-grain processed foods is a cause for concern. DON is the most prevalent and severe trichothecene toxin in these foods. However, D3G, NIV, and 3AcDON often coexist, and their harmful effects may be added. Currently, relevant standards are not perfect, and the contamination situation is underestimated. It is recommended to strengthen the monitoring of trichothecene toxin contamination in whole-grain raw materials and improve the processing technology of prepared foods. In addition, it is very necessary and urgent to formulate detailed maximum limits for related toxins in these foods.

This study used the recommended daily intake value of whole grains for adults in the Chinese Dietary Guidelines as the food factor (F value) for estimating exposure. This assumption is based on the following considerations: First, whole-grain prepared foods are one of the important sources of whole-grain intake in the diet; second, in the absence of actual survey data, the recommended value in the dietary guidelines can be used as a reference benchmark for assessing potential exposure risks [62]. However, it should be noted that it is crucial to articulate that such risk quantifications incorporate several assumptions. These include standardized consumption data, uniform absorption rates, and constant daily intake over time, which may not capture the nuances of population-specific dietary habits or acute consumption patterns. Moreover, the TDI value for DON is itself based on certain toxicological assumptions that might not encompass all subpopulations’ sensitivities. Despite these inherent uncertainties, employing this WHO-recommended approach provides an initial, albeit approximate, framework to gauge potential health risks and guide preventative strategies. The assessment acts as an impetus for more nuanced, targeted studies and supports the necessity for stringent control measures to mitigate DON contamination in whole-grain prepared foods. In the future, it will be necessary to conduct a nationwide consumption survey of whole-grain prepared foods to obtain more accurate food factor data to better assess the exposure level and health risks of the population.

## Figures and Tables

**Table 1:** Analytical parameters for the determination of 7 B type trichothecenes in ESI (–) using the UPLC–MS/MS method.

Analytes	Adduct	Retention Time (min)	*m*/*z*	Precursor Ion	Collision Energy (V)	Product Ion	Collision Energy (V)
DON	[M+CH_3_COO]^−^	7.43	355.1	295.1	−14	59.2	−50
D3G	[M+CH_3_COO]^−^	7.25	517.1	427.1	−30	457.1	−20
DOM	[M+CH_3_COO]^−^	8.34	339.1	249.1	−15	59.1	−50
FusX	[M+CH_3_COO]^−^	8.60	413.3	262.9	−22	59.1	−50
NIV	[M+CH_3_COO]^−^	6.40	371.1	281.1	−30	59.0	−46
3AcDON	[M+CH_3_COO]^−^	10.46	397.3	337.1	−13	307.2	−40
15AcDON	[M+CH_3_COO]^−^	10.33	397.3	59.0	−40	337.1	−9

**Table 2:** Linearity data, LOD, LOQ, and matrix effect (ME, %) for tested compounds by SPE–UPLC–MS/MS.

B-Type C-TCNs	Matrixs	Calibration Range (μg/kg)	Linear Equation	Correlation Coefficient (r)	LOD (μg/kg)	LOQ (μg/kg)	ME (%)
DON	Ready–to–eat brown rice	1–1000	y = 28,048x + 111,732	0.9991	0.4	1	−6.4
	Oatmeal		y = 27,171x + 137,284	0.9991	0.4	1	−10.6
	Whole wheat bread		y = 28,249x + 98,763	0.9998	0.3	0.9	−8.1
D3G	Ready–to–eat brown rice	0.2–200	y = 18,775x − 987	0.9996	0.06	0.2	−14.0
	Oatmeal		y = 17,106x − 1211	0.9993	0.05	0.15	−9.3
	Whole wheat bread		y = 17,614x − 373	0.9995	0.05	0.15	−10.0
3AcDON	Ready–to–eat brown rice	1–1000	y = 25,092x + 52,203	0.9992	0.2	0.8	−2.5
	Oatmeal		y = 24,956x + 34,251	0.9994	0.25	0.8	−6.6
	Whole wheat bread		y = 24,278x + 19,342	0.9994	0.3	1	−5.5
15AcDON	Ready–to–eat brown rice	2–2000	y= 12,969x + 10,982	0.9993	0.5	1.5	5.4
	Oatmeal		y= 13,311x + 41,537	0.9996	0.6	2	3.4
	Whole wheat bread		y= 12,510x + 38,762	0.9997	0.5	1.5	4.5
DOM–1	Ready–to–eat brown rice	1–1000	y = 22,102x + 12,324	0.9996	0.4	1	1.6
	Oatmeal		y = 24,397x + 10,981	0.9998	0.3	1	2.0
	Whole wheat bread		y = 22,274x + 8976	0.9993	0.4	1	2.6
FusX	Ready–to–eat brown rice	1–1000	y = 7115x + 3791	0.9998	0.2	0.8	−3.1
	Oatmeal		y = 6113x + 3429	0.9997	0.2	0.8	−6.2
	Whole wheat bread		y = 6202x + 2167	0.9995	0.3	1	−3.6
NIV	Ready–to–eat brown rice	1–1000	y = 10,810x + 8035	0.9994	0.3	1	−9.3
	Oatmeal		y = 9879x + 8035	1.0000	0.3	1	−12.8
	Whole wheat bread		y = 9976x + 8035	0.9994	0.3	1	−12.2

**Table 3:** Dietary exposure risk assessment of trichothecene mycotoxins in grain processed foods in the Chinese population.

Whole Grain Pre-Processed Foods	Type B Trichothecenes	C (μg/kg)	TDI (μg/kg·day·bw)	EDI (μg/kg·day·bw)	HQ (%)	HI (%)
Ready-to-eat brown rice	DON	45.60	1	0.0760	7.600	9.510
	D3G	2.24	0.3	0.0037	1.242	
	NIV	2.06	0.7	0.0034	0.490	
	3AcDON	0.67	1	0.0011	0.112	
	15AcDON	0.25	1	0.0004	0.042	
	FusX	0.10	0.7	0.0002	0.024	
Oatmeal	DON	260.36	1	0.4339	43.393	60.419
	D3G	24.95	0.3	0.0416	13.862	
	NIV	3.93	0.7	0.0066	0.936	
	3AcDON	12.28	1	0.0205	2.046	
	15AcDON	0.96	1	0.0016	0.159	
	FusX	0.10	0.7	0.0002	0.024	
Ready-to-eat oatmeal	DON	133.93	1	0.2232	22.321	27.010
	D3G	6.86	0.3	0.0114	3.808	
	NIV	1.27	0.7	0.0021	0.303	
	3AcDON	3.03	1	0.0050	0.504	
	15AcDON	0.30	1	0.0005	0.050	
	FusX	0.10	0.7	0.0002	0.024	
Oatmeal cookies	DON	55.86	1	0.0931	9.310	11.490
	D3G	2.89	0.3	0.0048	1.606	
	NIV	1.29	0.7	0.0022	0.308	
	3AcDON	1.15	1	0.0019	0.192	
	15AcDON	0.30	1	0.0005	0.050	
	FusX	0.10	0.7	0.0002	0.024	
Whole-wheat bread	DON	279.51	1	0.4659	46.585	57.242
	D3G	13.83	0.3	0.0230	7.681	
	NIV	3.59	0.7	0.0060	0.854	
	3AcDON	8.88	1	0.0148	1.480	
	15AcDON	3.43	1	0.0057	0.572	
	FusX	0.30	0.7	0.0005	0.071	
Whole-wheat noodles	DON	354.05	1	0.5901	59.009	71.100
	D3G	14.47	0.3	0.0241	8.041	
	NIV	5.21	0.7	0.0087	1.241	
	3AcDON	12.13	1	0.0202	2.022	
	15AcDON	4.42	1	0.0074	0.736	
	FusX	0.21	0.7	0.0004	0.051	
Whole-wheat dumpling wrapper	DON	590.77	1	0.9846	98.462	136.408
	D3G	52.70	0.3	0.0878	29.277	
	NIV	10.84	0.7	0.0181	2.580	
	3AcDON	31.40	1	0.0523	5.234	
	15AcDON	3.88	1	0.0065	0.647	
	FusX	0.88	0.7	0.0015	0.209	

Note: Whole-grain prepared foods consumption is 100 g/d. C: mean concentration of mycotoxin in samples. Mean body weight is 60 kg. Mycotoxin intake = (Whole-grain prepared foods consumption × C)/(mean body weight × 1000). HQ (Hazard Quotient, %) = mycotoxin intake × 100/TDI. HI (Hazard Index, %) = ∑HQ.

## Data Availability

All data used in this work is available either within the article or in the Supporting Information File.

## Supplementary Materials

The following supporting information can be downloaded at: https://www.mdpi.com/article/10.3390/toxics12050336/s1, Table S1: A summary of B-type trichothecenes detection in grains via LCMS/MS over the last decade. Table S2: Accuracy and precision data for determination of 7 mycotoxins at three levels in one day (*n* = 6) and three distinct days (*n* = 18). Table S3: The occurrence of 7 TCNs in 160 Whole grain prepared foods samples from 8 food categories collected in Chinese market.

## Abbreviations

Solid phase extraction (SPE), restricted-access media (RAM), trichothecenes (TCNs), deoxynivalenol (DON), 3–acetyldeoxynivalenol (3AcDON), 15–acetyldeoxynivalenol (15AcDON), deoxynivalenol 3–glucuronide (D3G), de-epoxy-deoxynivalenol (DOM–1), fusarenon–X (FusX), nivalenol (NIV), ultra-high-performance liquid chromatography-tandem mass spectrometry (UHPLC–MS/MS), limit of detection (LOD), limit of quantification (LOQ).
