# Supplementary material for: Restricted-Access Media Column Switching Online Solid-Phase Extraction UHPLC–MS/MS for the Determination of Seven Type B Trichothecenes in Whole-Grain Preprocessed Foods and Human Exposure Risk Assessment
Source: Toxics. 2024 May 6;12(5):336. doi: 10.3390/toxics12050336 (PMC11126074; doi:10.3390/toxics12050336)
Supplement: Supplementary file 1 [file toxics-12-00336-s001.zip › toxics-2970983-supplementary.pdf]

Restricted–access media column switching online solid–phase extraction UHPLC–MS/MS for the determination of 7 type B trichothecenes in whole grain pre–processed foods and human exposure risk assessment

Xiao Ning[a, b], *Yongli Ye*[a], *Jian Ji*[a], *Yanchun Hui*[c], *Jingyun Li*[b],*Po Chen*[b], *Shaoming Jin*[b], *Tongtong Liu*[b],*Yinzhi Zhang*[a], *Jin Cao*[b,*] *and Xiulan Sun*[a,*]

[a] School of Food Science and Technology, International Joint Laboratory on Food Safety, Synergetic Innovation Center of Food Safety and Quality Control, Jiangnan University, Wuxi, Jiangsu 214122, China

[b] Key Laboratory of Food Quality and Safety for State Market Regulation, National Institute of Food and Drug Control, Beijing 100050, China

[c] Sanyo Fine Trading Co., LTD., 100176, China

* Correspondence: sxlzzz@jiangnan.edu.cn (X.S.); caojin@nifdc.org.cn (J.C.); Tel.: +86-0510-8591-2330 (X.S.); +86-010-6709-5070 (J.C.)

2. Methods

2.1 Linearity and matrix effect

Linearity was evaluated using matrix–matched calibration curves. In this experiment, blank samples of each food matrix were spiked with a sufficient amount of a series of standard solutions containing seven concentrations. Each level was injected three times using LC–MS/MS. Matrix effects (ME) can influence the accuracy and precision of the analytical method, leading to incorrect analytical results. ME was evaluated using matrix–matched calibration and standard solution calibration in pure solvent at the same concentration level. ME (%) was calculated based on the slopes of the calibration plots for each mycotoxin in the neat standard solution and the spiked blank sample extract using the formula: ME (%) = 100 – (100 × slope of spiked blank sample extract/slope of neat standard solution). ME (%) values within the range of –20% and 20% were considered tolerable, while ME values exceeding the above range indicated critical matrix effects [9].

2.2. LOD and LOQ

The sensitivity of the method was verified by determining the LOD and LOQ for each mycotoxin. LOD and LOQ were defined as the minimum concentrations of each respective mycotoxin with signal–to–noise ratios of 3 and 10, respectively.

2.3. Selectivity

The selectivity of the method was investigated by analyzing blank samples and blank samples spiked with the target mycotoxins. This was done by monitoring the characteristic MRM transitions for each mycotoxin at the corresponding retention times and matching the relative response ratios of the quantitation and confirmation channels.

2.4. Accuracy and precision

The accuracy of the established method was verified by evaluating the recovery of blank samples spiked with mycotoxins at different concentrations. Intra–day precision was assessed for all concentration levels of each compound on the same day, three times in total (n = 3), and the results were expressed as the average relative standard deviation (% RSD). Inter–day precision was determined using the average of three concentration levels (low, medium, and high) analyzed in triplicate (n = 3) on different dates, and the results were expressed as the average % RSD.

Table S1 A summary of B-type trichothecenes detection in grains via LC-MS/MS over the last decade

No.	Matrixs	Analytes	LOD (μg/kg)	LOQ (μg/kg)	Positive rate %（Number of positive samples/Total samples）	Reference
1	otas	DON	0.12	0.38	50%(n=10)	[23]
2	Corn	DON	6.7	11.9	90%(n=10)	[9]
		FUSX	4.0	8.3	0(n=10)	
		3ADON	1.9	5.3	0(n=10)	
		15ADON	3.6	7.3	60%(n=10)	
3	Maize	DON	0.11	0.36	/	[24]
		NIV	0.14	0.45	/	
		3ADON	0.16	0.64	/	
		15ADON	1.8	6.5	/	
		FUSX	0.15	0.54	/	
		D3G	0.02	0.11	/	
4	rice	DON	0.14	0.43	35%(n=20)	[25]
		3AcDON	0.15	0.45	-	
		15AcDON	0.10	0.29	-	
		NIV	0.42	1.26	30%(n=20)	
5	Wheat, rice and maize	DON				[26]
		NIV	0.02-2	0.06-6		
		3AcDON				
		15AcDON				
6	Wheat, corn	15AcDON	-	200	46%(n=200)	[27]
		DON	-	200	61%(n=200)	
		FUSX	-	200	0%(n=200)	
		NIV	-	200	18%(n=200)	
		3AcDON	-	200	7%(n=200)	
		FUSX	-	200	39%(n=200)	
		D3G	-	20	0%(n=10)	
7	sorghum	15AcDON	31	62.5	0%(n=10)	[28]
		DON	12.5	25	0%(n=10)	
		FUSX	31	62.5	0%(n=10)	
		NIV	50	100	0%(n=10)	
		3AcDON	15.6	31	50%(n=10)	

Table S2 Accuracy and precision data for determination of 7 mycotoxins at three levels in one day (n = 6) and three distinct days (n = 18).

Analytes	Spiked level （μg/kg）	Ready-to-eat whole grain multigrain rice				Oatmeal				Whole wheat bread			
		Measured value （μg/kg）	Accuracy (n= 6, %)	Intra-day (n= 6, %)	Inter-day (n = 36, %)	Measured value （μg/kg）	Accuracy (n= 6, %)	Intra-day (n= 6, %)	Inter-day (n = 36, %)	Measured value （μg/kg）	Accuracy (n= 6, %)	Intra-day (n= 6, %)	Inter-day (n = 36, %)
DON	1	0.9	101.9	3.4	9.1	0.8	93.2	3.9	8.7	0.9	98.0	2.5	9.5
	10	8.8	96.3	1.8	3.3	8.4	90.8	4.1	7.9	9.8	99.7	3.2	4.4
	800	712.0	101.9	3.4	7.8	749.6	94.4	3.7	4.3	746.4	98.7	1.4	6.0
D3G	0.2	0.2	101.3	3.7	4.4	0.2	91.8	4.1	7.8	0.2	98.1	2.5	8.6
	2	2.0	97.8	2.1	9.3	1.7	92.9	3.2	5.5	1.8	93.7	3.2	6.9
	160	151.7	101.9	1.7	7.1	150.1	95.8	3.2	7.3	156.3	98.9	2.7	4.4
3AcDON	1	1.0	97.0	3.2	8.2	0.9	93.5	3.8	6.2	1.0	93.7	2.5	6.8
	10	9.1	99.2	3.0	5.0	8.9	92.7	3.4	6.8	9.3	97.6	1.5	8.8
	800	678.4	94.8	3.4	5.4	687.2	90.7	2.8	5.2	757.6	95.8	3.4	7.8
15AcDON	2	1.8	96.5	3.2	9.1	1.9	95.8	4.1	5.8	1.8	93.6	1.5	8.4
	20	18.3	94.8	2.4	9.1	18.9	93.9	3.2	4.1	19.1	100.7	2.3	9.9
	1600	1516.8	95.6	2.0	4.1	1523.2	96.8	4.1	6.3	1486.4	99.0	1.7	11.7
DOM	1	0.9	95.0	3.1	4.6	1.0	96.6	4.3	8.0	1.0	99.4	2.7	9.2
	10	10.1	99.4	2.0	9.7	8.6	91.6	3.9	8.3	9.0	106.6	1.7	7.6
	800	729.6	98.9	2.4	9.7	683.2	96.6	2.9	5.9	732.0	94.7	1.6	3.7
FusX	1	1.0	100.7	2.0	5.8	0.9	95.9	3.0	9.0	1.0	95.1	2.0	4.2
	10	9.9	95.7	2.6	6.2	8.9	94.5	3.4	7.4	9.5	93.7	2.7	7.3
	800	756.0	101.7	1.7	8.0	665.6	90.3	3.5	4.6	764.8	101.9	2.7	8.1
NIV	1	0.9	96.9	3.0	9.3	0.9	92.4	4.1	6.1	0.9	97.8	2.1	4.7
	10	8.9	96.6	2.8	7.6	9.4	93.1	4.1	4.7	9.9	98.2	2.4	6.6
	800	784.0	96.4	1.7	4.2	732.8	91.7	2.9	4.2	830.4	94.9	2.8	3.8

Table S3 The occurrence of 7 TCNs in 160 Whole grain prepared foods samples from 8 food categories collected in Chinese market

Analytes		Infant whole grain rice flour	Ready-to-eat brown rice rice	Oatmeal	Ready-to-eat oatmeal	Oatmeal cookies	Whole wheat bread	Whole wheat noodles	Whole wheat dumpling wrapper	Whole grain prepared foods
DON	Positive samples/ Samples analyzed (n/n)	4/20	12/20	18/20	15/20	8/20	17/20	19/20	19/20	112/160
	Range (μg/kg)	2.4-33.1	10.3-169.8	50.6-719.1	20.9-203.8	18.7-214.2	49.6-583.2	88.5-701.9	60.3-1685.2	2.4-1685.2
D3G	Positive samples/ Samples analyzed (n/n)	0/20	3/20	7/20	4/20	3/20	4/20	5/20	9/20	35/160
	Range (μg/kg)	ND	5.7-25.6	1.4-147.0	0.9-97.9	0.6-37.3	2.7-131.5	4.1-118.0	3.9-208.1	0.6-208.1
NIV	Positive samples/ Samples analyzed (n/n)	0/20	6/20	8/20	7/20	3/20	10/20	13/20	14/20	61/160
	Range (μg/kg)	ND	1.7-11.1	2.6-19.4	1.6-5.0	ND	2.3-12.6	3.0-14.7	2.0-28.6	1.6-28.6
3AcDON	Positive samples/ Samples analyzed (n/n)	2/20	2/20	6/20	8/20	5/20	6/20	9/20	11/20	49/160
	Range (μg/kg)	1.3-4.9	2.2-9.4	3.4-76.1	2.6-13.7	1.9-6.3	6.1-55.2	5.2-65.2	9.9-90.3	1.3-90.3
15AcDON	Positive samples/ Samples analyzed (n/n)	0/20	0/20	2/20	0/20	0/20	3/20	3/20	6/20	13/160
	Range (μg/kg)	ND	ND	2.9-10.8	ND	ND	4.7-39.1	4.0-47.2	6.6-23.8	2.9-47.2
FusX	Positive samples/ Samples analyzed (n/n)	0/20	0/20	0/20	0/20	0/20	1/20	1/20	3/20	5/160
	Range (μg/kg)	ND	ND	ND	ND	ND	3.1	1.4	2.8-17.9	1.4-17.9
DOM-1	Positive samples/ Samples analyzed (n/n)	0/20	0/20	0/20	0/20	0/20	0/20	0/20	0/20	0/160
	Range (μg/kg)	ND	ND	ND	ND	ND	ND	ND	ND	ND

*ND：non-detection; non-detection was treated as LOD/2 during the calculation of the arithmetic mean
